# Dissociating the white matter tracts connecting the temporo-parietal cortical region with frontal cortex using diffusion tractography

**DOI:** 10.1038/s41598-020-64124-y

**Published:** 2020-05-18

**Authors:** Elise B. Barbeau, Maxime Descoteaux, Michael Petrides

**Affiliations:** 1 Cognitive Neuroscience Unit, Department of Neurology and Neurosurgery, Montreal Neurological Institute, McGill University, 3801 University, Montreal, QC H3A 2B4 Canada; 2 Center for Research on Brain, Language and Music (CRBLM), 3640 de la Montagne Montreal, Montreal, QC H3G 2A8 Canada; 3 Department of Psychology, McGill University, 2001 Avenue McGill College, Montreal, QC H3A 1G1 Canada; 4 Sherbrooke Connectivity Imaging Lab (SCIL), Computer Science Department, Université de Sherbrooke, 2500 Boulevard de l’Université, Sherbrooke, QC J1K 0A5 Canada

**Keywords:** Neuroscience, Brain

## Abstract

Three major white matter pathways connect the posterior temporal region and the adjacent inferior parietal lobule with the lateral frontal cortex: the arcuate fasciculus (AF), and the second and third branches of the superior longitudinal fasciculus (SLF II and SLF III). These pathways are found also in nonhuman primate brains where they play specific roles in auditory and spatial processing. The precise origin, course, and termination of these pathways has been examined in invasive tract tracing studies in macaque monkeys. Here we use this prior knowledge to improve dissections of these pathways *in vivo* in the human brain using diffusion Magnetic Resonance Imaging (MRI) tractography. In this study, the AF, originating from the posterior temporal cortex, has been successfully separated from the SLF II and SLF III tracts originating from the angular and supramarginal gyri of the inferior parietal lobule, respectively. The latter two pathways, i.e. SLF II and SLF III, have also been clearly separated from each other. Furthermore, we report for the first time in the human brain the dorsal branch of the AF that targets the posterior dorsolateral frontal region. These improved dissection protocols provide a solid basis for exploring the respective functional roles of these major fasciculi.

## Introduction

The arcuate fasciculus (AF) is the white matter tract *arching* (hence the name *arcuate*) around the end of the Sylvian fissure to connect the posterior temporal region of the brain with specific parts of the frontal lobe. In the language dominant hemisphere, it connects the posterior temporal region involved in the comprehension of language (often referred to as Wernicke’s area) with the inferior frontal gyrus (IFG) where Broca’s area lies, i.e. the key region for language production. Broca’s area comprises two distinct morphological structures, the pars opercularis where cytoarchitectonic area 44 is located and the pars triangularis where area 45 lies^1,2^. Lesions of the fibers of the left AF disconnecting the posterior temporal language region from the IFG are considered to result in conduction aphasia^3^, a syndrome characterized by an impairment in the repetition of speech, including phonological paraphasias and impaired learning and attention to speech (^4^, for a review). The problem with drawing conclusions regarding the functional contributions of specific white matter tracts is that other tracts may be coursing adjacent to the tract of interest. For instance, in the fronto-parietal white matter, *parallel to the AF* course fibers belonging to the two branches of the superior longitudinal fasciculus (SLF) that originate from distinct parts of the inferior parietal lobule: SLF II from the angular gyrus and SLF III from the supramarginal gyrus. Accurate dissections of these adjacent white matter tracts and distinguishing them from the AF remain of paramount importance if we wish to examine their distinct functional contributions.

The first description of the AF came from post-mortem dissections in which the arching fibers around the posterior end of the Sylvian fissure could clearly be observed. Dejerine^5^ was among the first to describe this tract anatomically as “temporo-occipito-frontal” connections, a vague terminology which emphasizes the fact that the exact origin and termination of the fibers could not be demonstrated, a common issue with post-mortem dissections. More recent work using diffusion Magnetic Resonance Imaging (MRI) to reconstruct this tract has provided various *in vivo* dissection protocols of the AF in the human brain^6–8^. These protocols using diffusion tensor imaging deterministic tractography have expanded the definition of the AF beyond the classical arching fibers around the Sylvian fissure that link posterior temporal cortex with IFG. The classical arcuate (i.e. arching) fasciculus is referred to as the “long segment” in one protocol^6,7^ and other nearby tracts are included as part of the arcuate system, such as an “anterior segment” connecting the inferior parietal region (which is referred to as Geschwind’s region) with the IFG (Broca’s region)^7,9^. The fibers linking the inferior parietal lobule (IPL) with the frontal cortex are, of course, fibers of the classic superior longitudinal fasciculus (SLF). In other studies, the SLF terminology is used for a group of tracts that also includes the AF and the middle longitudinal fasciculus that connects inferior parietal cortex with temporal cortex [e.g.^10^]. This variability in the terminology used to describe the fronto-parieto-temporal tracts, together with differences in the definition of what brain areas each tract connects contributes to a confusion in this area of research. In the macaque monkey in which the precise axonal origin, course, and terminations of white matter tracts can be established by the use of invasive anatomical methods, the SLF from the IPL was divided into SLF II originating from the posterior IPL (homologue of the angular gyrus) and terminating in a specific set of frontal areas and another distinct branch, SLF III, originating from the anterior IPL (homologue of the supramarginal gyrus) and targeting another distinct set of frontal areas^11^. Thus, the AF (the posterior temporo-frontal arching pathway) and the two tracts (SLF II and SLF III) coursing in parallel with it in the fronto-parietal white matter link specific cortical regions and failure to dissect them separately makes it impossible to examine their distinct functional roles. Recent advances in image processing algorithms for diffusion tractography permit improved modeling of crossing fibers and allow further investigation of these white matter tracts [e.g.^12,13^].

Note that just as classical white matter dissections^14,15^, diffusion MRI tractography cannot establish the precise cortical origins and terminations of particular white matter tracts, and other limitations have to be considered^16^. For example, it is not possible to determine the direction of transmission of white matter fibers or whether connections from one brain area to another are *monosynaptic via a specific fasciculus* or a polysynaptic series of connections [see^17^ for a review]. Technical limitations also make it difficult to reconstruct tracts where crossing fibers are predominant or where more than one tract course in parallel adjacent to each other in both classical white matter dissections and diffusion MRI^17^.

In light of the limitations in studying white matter tracts in the human brain both with the classical white matter dissections and modern diffusion MRI, the use of invasive anatomical tracers in the macaque monkey remains the gold standard for examining *precise* anatomical connectivity in the primate cortex. The injection of tracers, such as radioactively labeled amino acids, into a specific cortical area (i.e. the injection is restricted *within* the cortical grey matter) permits the tracing of the exact course, direction, and termination in distant cortical areas of the axons originating from the injected cortical region. Furthermore, the terminations within particular distant cortical areas of the axons that originated in the particular cortical area injected with radioactively labeled amino acids can be confirmed (in another group of animals) by the injection of retrograde tracers (e.g. fast blue) in the cortical termination sites and subsequent examination of the region of origin of the studied pathway for cell bodies labeled with the retrograde tracer (e.g. fast blue) used. Thus, in macaque monkey tracing studies, there is a) precise tracking of the axonal course of a particular pathway, including its terminations in distant cortical areas and b) confirmation of the precise cortical location of the cells of origin of the white matter pathway under study by the injection of retrograde tracers at its distant target terminations.

It is important to note that invasive axonal tracing in the macaque monkey has clearly separated the AF originating in the posterior temporal region from the adjacent white matter fibers that connect IPL with the frontal cortex, i.e. the classic superior longitudinal fasciculus (SLF) branches II and III. Furthermore, although all descriptions and reconstructions of the AF in the human brain refer only to connections of the posterior temporal region with the inferior frontal gyrus, precise invasive tract tracing in the macaque monkey has shown that, in addition to the classic inferior frontal connections of the AF, there is also a *dorsal* branch that connects the posterior temporal region involved in the processing of high-level auditory information with the posterior dorsolateral frontal region^18,19^ which is a hub for attentional control (area 8A)^20^. The dorsal branch of the AF has never been reported in the human brain because the focus was always on the connections of the posterior temporal region with the IFG (Broca’s area). Thus, another major aim of the present investigation has been to examine whether the dorsal branch of the AF clearly demonstrated in the macaque monkey can also be identified in the human brain. This is critical because if indeed the dorsal branch exists in the human brain, it would be a pathway allowing communication between the dorsolateral frontal attention control region (area 8A) and the posterior temporal region involved in the processing of high-level auditory information, primarily verbal auditory in the language-dominant left hemisphere and nonverbal auditory (e.g. music) in the non-dominant right hemisphere.

The present study used critical information from experimental anatomical studies in macaque monkeys, as well as earlier *in vivo* diffusion MRI findings and resting state connectivity data in the human brain, to improve dissections *in vivo* in the human brain of 1) the AF (i.e. the *arching* temporo-frontal fiber tract), 2) the SLF II and III tracts originating in the IPL, and 3) to search for the dorsal branch of the AF in the human brain, based on precise anatomical landmarks which are necessary to improve the accuracy and sensitivity of future tractography^16,21^. The functional properties of a white matter tract are the result of the interactions between the distinct regions that it connects. Failure to separate accurately the white matter pathways means that it is not possible to examine their distinct functional contributions.

Thus, the aim of the present study is to provide specific guidelines to dissect the AF separately from the two adjacent pathways that course parallel to it in the fronto-parietal white matter, i.e. the SLF III originating from the supramarginal gyrus and the SLF II from the angular gyrus, and also to examine the predicted dorsal branch of the AF in the human brain that had never been demonstrated before. The distinction between these different tracts is not clearly made in the current tractography literature that tends to view these distinct white matter fibers connecting the frontal to posterior temporal and adjacent inferior parietal areas as a unified system based on the fact that these tracts course in parallel in the brain’s fronto-parietal white matter stem and are thus difficult to reconstruct with current diffusion MRI methodology.

The present study also used seed-based resting state connectivity to support the distinct patterns of regional correlations in intrinsic brain activity between the two different inferior parietal lobule regions (i.e. angular and supramarginal gyri), as well as the posterior temporal region, and their respective frontal targets. More generally, this study aims to provide improved protocols for tractography dissections using Diffusion Tensor Imaging (DTI) and also High Angular Resolution Diffusion Imaging (HARDI) probabilistic tractography of the white matter pathways that originate from the posterior temporal region and adjacent inferior parietal lobule based on specific knowledge of what cortical areas each pathway connects. Thus, this research can provide the basis for more uniform and accurate future correlation analyses of properties of these tracts with behavioral language and non-linguistic measures (e.g., musical processing in the posterior superior temporal region and spatial processing in the posterior parietal region of the non-dominant hemisphere) that can refine our understanding of the distinct functional contributions of these tracts and the brain regions they connect.

## Materials and Methods

### Participants

50 right-handed healthy volunteers (mean age 23.9 years, range 18–34 years, 24 females) were included in this study. All participants scored within the normal range of intellectual functioning on the Matrix Reasoning subtest of the Wechsler Adult Intelligence Scale (group mean score 19.4, range 9–25). The exclusion criteria were any neurological, psychiatric or other medical condition and medication known to affect brain structure or function. A financial compensation was offered to the subjects and all participants in this study gave their written informed consent and all experimental protocols were performed in accordance with the ethical standards of the Research Ethics Board at the Montreal Neurological Institute, McGill University and with the Declaration of Helsinki.

### MRI image acquisition

Magnetic resonance imaging data were acquired on a Siemens 3 T Tim Trio Scanner using a 32-Channel head coil at the McConnell Brain Imaging Center of the Montreal Neurological Institute. The scanning session included the acquisition of diffusion-weighted magnetic resonance imaging data (73 slices, TR = 10000 ms, TE = 90 ms, 2mm^3^ voxels, slice thickness of 2 mm, directions=64, b = 1000 s/mm^2^), as well as a T1-weighted structural scan obtained with an MPRAGE sequence (192 slices, TR = 2300 ms, TE = 2.98 ms, flip angle = 9°). For the Resting State functional magnetic resonance imaging (fMRI) sequence, 38 3.5-mm-thick transverse slices (TR: 2260 ms, TE: 30 ms, 3.5 mm^3^ voxels, matrix size: 64 × 64, FoV 224 mm, flip angle 90 ^o^) were acquired.

### Data analysis

#### Diffusion weighted and T1-weighted imaging analysis

Susceptibility-induced distortions were corrected through the application of FSL/TOPUP and eddy current corrections^22^. The T1w image was processed using the Desikan-Killiany FreeSurfer Atlas (http://surfer.nmr.mgh.harvard.edu/) to obtain cortical parcellations of interest for each participant’s brain^23^. The brain extraction tool FSL Fast was used to obtain partial volume estimation (PVE) maps for white matter, grey matter, and cerebrospinal fluid from the T1w images^24^.

##### Probabilistic HARDI tractography

Reconstruction of fiber orientation distribution functions (fODFs)^25^ was performed with maximal spherical harmonics order 8 and constrained spherical deconvolution using Dipy^26^, followed by anatomically-constrained informed particle-filtering probabilistic tractography^27^, seeding from the union of the white matter and white matter/grey matter interface masks, using 10 seeds per voxel leading to tractograms of approximately 2 million streamlines and 20–200 mm in length for each participant^26^. The step size used for tractography was 0.5 mm with a second order runge-kutta integration, 20 degree angular threshold, a minimum radius of curvature of 1 mm, and fODF amplitude cutoff at 0.1, as recommended by Girard and colleagues^27^. All streamlines detected to make a 300 degree loop onto themselves were removed from tractograms^28–30^. Tractograms were subject to streamline fiber compression with a 0.2 tolerance rate to reduce the file size by a factor of 10^31,32^.

##### Probabilistic DTI tractography

The classical diffusion tensor (DT) model approach remains the most widely used method to dissect the language tracts because of their simplicity and to avoid the false positive problems that come with more complex and permissive models^33^. Because one of the objectives of the present investigation was to provide guidelines for *in vivo* tract dissections regardless of the tractography algorithm used, we also processed the same datasets using a DTI pipeline. DTI tractography was launched with the MRtrix3 “Tensor_Prob” option^34^. Default parameters and the exact same seeding mask and same total number of reconstructed streamlines as in the HARDI protocol were used for every participant in order to mimic as closely as possible the HARDI approach described above. This approach also allows for a comparison of reconstruction success for our three tracts of interest with the different methods in line with Bain and colleagues^33^ who demonstrated that the laterality indices of the AF varied according to the tractography method used. The tract reconstructions with both the DTI and HARDI approaches were carried out on a subsample of the participants (the first 35 participants out of 50).

#### Tract dissections

##### Arcuate fasciculus:

**Figure 1:**
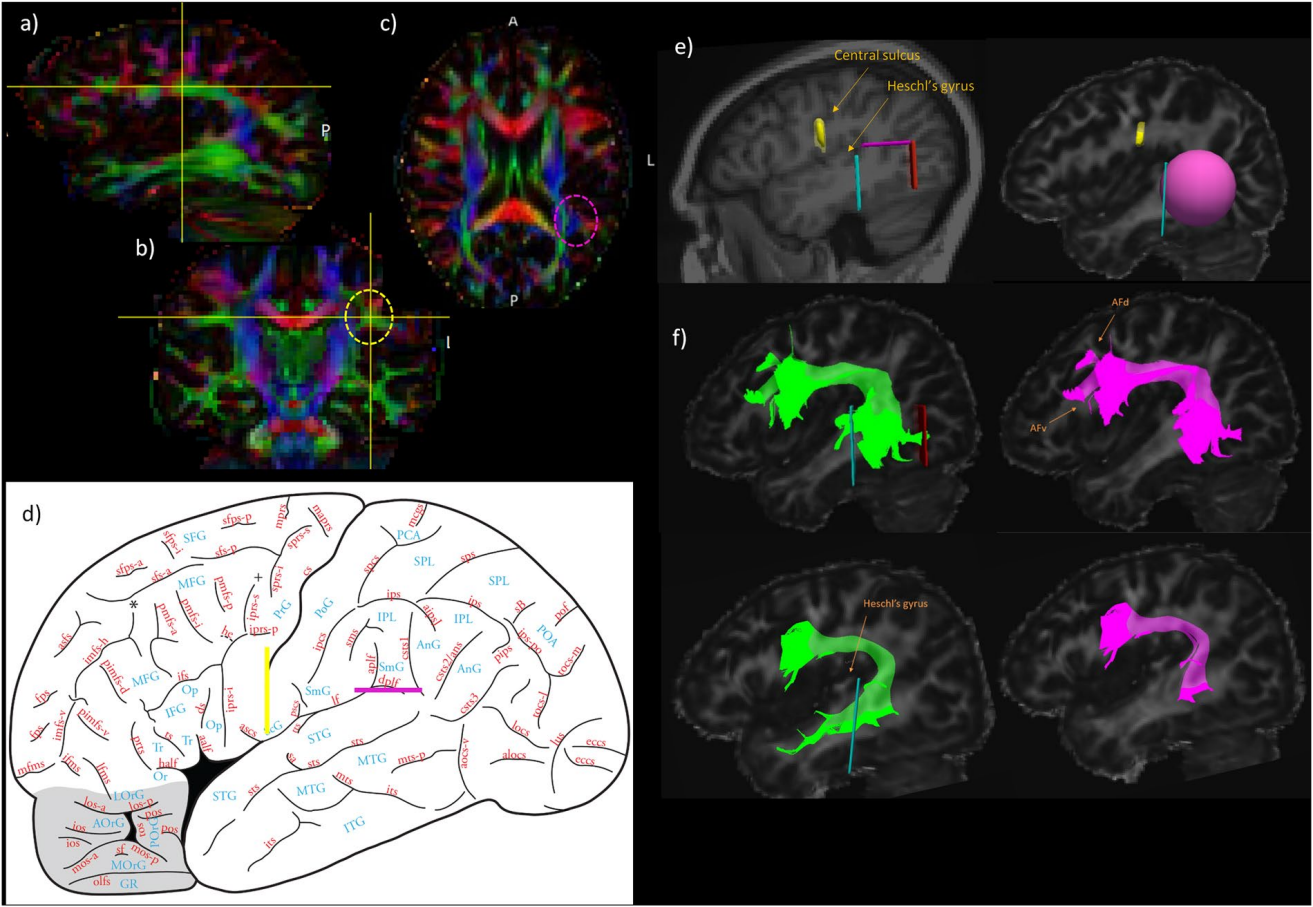
Inclusion Regions of Interest (ROIs) for dissection of the arcuate fasciculus (AF) shown on the FA colour map (**a**–**d**) on image adapted from^36^, page 19, with permission. (**e**) Inclusion ROIs for the dissection of the AF: yellow “fronto-parietal white matter way-point”, fuchsia sphere for the temporal ROI, and exclusion section planes (cyan and red) ROIs. (**f**) Example dissections of the AF in two participants (AFd: dorsal terminal branch, AFv: ventral terminal branch). In green, dissection of the Long segment of the AF following Catani’s method^6,7,9^ (without the exclusions). In pink, our dissection after the additional exclusion ROIs were applied.

First, on the FA-colour map, the C-shaped green tract was localised in the left hemisphere (Fig. 1a), and a first Region of Interest (ROI) was drawn in the coronal section immediately under the central sulcus, around the green triangle shape (see Fig. 1b,e: Yellow ROI), as in Wakana *et al*.^8^ and Catani and colleagues^6,7,9^. Because of the point-based streamline selection methods in Trackvis^32^, we drew the ROI on four adjacent coronal slices to ensure the selection of all streamlines passing within “any-part” of this ROI. A second inclusion ROI can be drawn in the axial view at the temporo-parietal junction (see Fig. 1d, fuchsia line). To do so, as in the Catani and the Wakana protocols, we localised the blue region where the vertical part of the AF is found (circled in fuchsia in Fig. 1c). However, in previous protocols, the level at which this ROI was placed was not specified. For example, Lopez-Barroso and colleagues^9^ mention that “the temporal ROI was placed on an axial slice” (*SI*, p.1), which could lead to variations in the dissection and leave out fibers ending in the superior temporal gyrus near the temporo-parietal junction if this ROI were to be placed too low. To avoid this potential problem, we used cortical anatomical landmarks to ensure that the fibers below the descending posterior ramus of the lateral fissure (dplf), which marks the border between the temporal and adjacent inferior parietal cortex, are included (Fig. 1d, fuchsia plane ROI, at Montreal Neurological Institute (MNI) z = 18). The fibers ending above this line are in the supramarginal gyrus of the inferior parietal lobule and, therefore, part of the SLF III rather than the AF. Fibers passing through both those ROIs (yellow and fuchsia) should be included in the AF reconstruction. Because we were using Trackvis (note the point-based problem mentioned above) and to restrict the streamlines to those terminating within the posterior part of the superior and middle temporal gyri, instead of a plane ROI, we used a sphere (radius of 20 mm) and centered around MNI coordinates x = −60, y = −45, z = 0 in the left hemisphere (Fig. 1e) and MNI coordinates x = 60, y = −43, z = −1 in the right hemisphere, as the temporal inclusion region which fits almost perfectly the shape of the lateral surface of the temporal lobe. The top of the sphere is at the dplf and the bottom at the inferior temporal sulcus (x = −19). The Trackvis condition “Either end” was used for this ROI. Furthermore, we added an exclusion ROI (which had not been used in previous dissection protocols) to ensure that only fibers originating from the posterior temporal cortex were included in the dissected AF. This exclusion ROI (Cyan ROI, Fig. 1e) was added in the coronal view, immediately posterior to the last slice where Heschl’s gyrus is visible. This decision was based on macaque monkey invasive tracing studies of the extent of the posterior superior temporal gyrus and adjacent superior temporal sulcal region that is linked via the AF [e.g.^18,35^]. Moreover, the use of the temporal sphere which, posteriorly, ends before the border of the temporal gyri with the occipital lobe ensures exclusion of any fibers originating in the occipital lobe (dissection protocols using planes as ROIs would necessitate an extra exclusion ROI at y = −38, see Red ROI example, Fig. 1e) which belong to the superior fronto-occipital fasciculus (SFOF; see scarlet red tract in [^36^, p. 171]). Additional exclusion ROIs were used on a case by case basis to exclude projection fibers directed to subcortical structures through the internal capsule. For all exclusion ROIs, the Trackvis condition ﻿“No part” was chosen. The same procedure was used to dissect the tracts in the right and left hemispheres. MNI coordinates for ROI placement in normalised space are provided in Fig. 3b.

##### Superior longitudinal fasciculus (SLF): second (SLF II) and third (SLF III) branches

**Figure 2:**
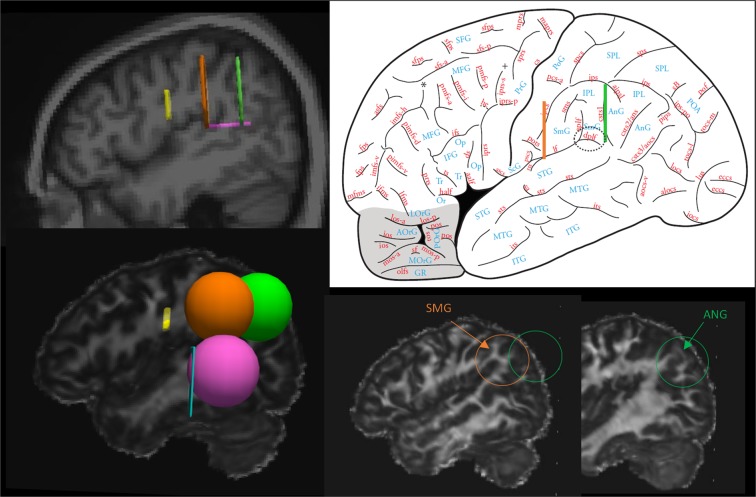
SLF III inclusion (yellow fronto-parietal white matter and orange sphere) and exclusion (green and fuchsia) limits and SLF II inclusion (yellow fronto-parietal white matter and green sphere) and exclusion (fuchsia sphere) ROIs. The descending posterior ramus of the lateral fissure (dplf), at the temporo-parietal junction, is circled (figure adapted from^36^, page 19, with permission). SMG (orange) and ANG (green) ROI inclusion spheres are shown as well as empty circles to illustrate the fit of the ROIs to the gyri of interest.

The dissection of the third branch of the superior longitudinal fasciculus (SLF III) started from the same yellow ROI below the central sulcus (Fig. 1b) and an inclusion ROI as a sphere placed in the supramarginal gyrus (orange sphere, Fig. 2). This sphere was 20 mm in radius and centered at MNI coordinates x = −55, y = −42, z = 36 and x = 55, y = −39, z = 37 for the left and right hemispheres, respectively. The bottom of the sphere ends at the dplf just above the temporal sphere. In order to include fibers originating and ending specifically in the supramarginal gyrus, i.e. the SLF III fibers, an exclusion ROI is placed in the angular gyrus (green sphere, Fig. 2; 20 mm radius centered at MNI [x = −41, y = −68, z = 38] and [x = 41, y = −65, z = 38]) from where the SLF II pathway is known to originate^11^. These ROIs in the angular gyrus in the left and right hemispheres are then used as inclusion ROIs to reconstruct the SLF II branch that connects the angular gyrus with the frontal lobe. Note that even when working with brains normalised to MNI stereotaxic space, the supramarginal and angular spheres must be adjusted in individual brains to the precise location of the relevant landmarks in each individual normalised brain to deal with individual subject variability. The coordinates provided indicate the location of the landmarks on the average Montreal Neurological Institute (MNI) brain (the MNI 152 template).

**Figure 3:**
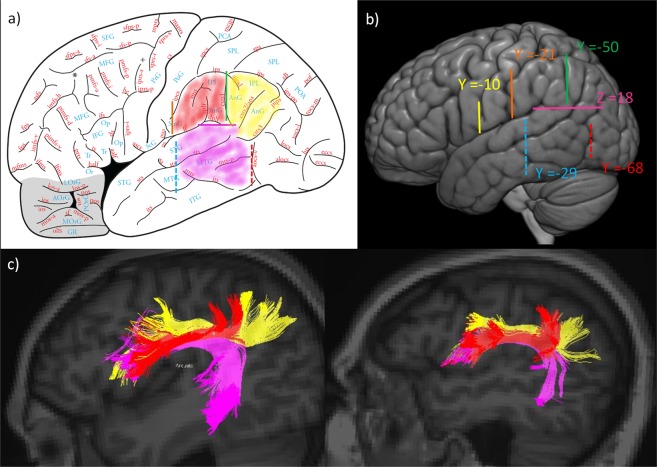
(**a**) Delineation of the acceptable regions of interest (ROIs) for the dissected pathways. The angular gyrus (AnG in yellow) for SLF II, the supramarginal gyrus (SmG in red) for SLF III, and the posterior part of the temporal lobe (in fuchsia) on figure adapted from^36^, page 19, with permission. (**b**) Critical locations for ROI placement with coordinates in Montreal Neurological Institute (MNI) stereotaxic space: Yellow ROI (Y = −10), Orange ROI (Y = −21), Green ROI (Y = −50), Fuchsia ROI (Z = 18), Red ROI (Y = −68), Cyan ROI (Y = −29). The same Y and Z coordinates are valid for the right hemisphere. (**c**) Final dissection using DTI of the SLF II (in yellow), the SLF III (in red), and the AF (in fuchsia) for two participants.

Alternatively, when using a DTI or deterministic approach, plane ROIs could be used instead of the spheres and these yield comparable reconstructions of the desired fasciculus. The SLF III dissections are achieved using a plane ﻿“Any part” ROI in a coronal section between the post-central gyrus and the supramarginal gyrus (orange ROI, Fig. 2). The temporo-parietal ROI (fuchsia) that was used as an inclusion for the AF is now used as an exclusion ROI for the SLF to ensure that only fibers connecting the inferior parietal lobule (and not the temporal lobe) are included in this dissection. In order to include fibers originating and ending specifically in the supramarginal gyrus, i.e. the SLF III fibers, an exclusion ROI is placed in another coronal section at the sulcus between the supramarginal gyrus and the angular gyrus (y = −50 on the MNI 152 template; green ROI, Fig. 2). This ROI is then used as an inclusion ROI for SLF II (connecting the angular gyrus to the frontal lobe). Note that even though these ROI selections were carried out in MNI stereotaxic space, the ROIs had to be adjusted according to the specific brain morphology of the different participants. Additional exclusions may have to be used on a case by case basis to exclude fibers diverging from the tract (e.g., fibers in the parietal lobe above the intraparietal sulcus or, for SLF II, fibers outside the angular gyrus). Figure 3 depicts the angular (yellow) and supramarginal (red) gyri of the inferior parietal lobule that are linked with specific areas of the IFG through the second and third branches of the superior longitudinal fasciculi (SLF II and SLF III), respectively, and the posterior temporal region that is linked via the ventral branch of the AF. MNI coordinates for all ROI placements in normalised space are provided in Fig. 3b, and Fig. 3c depicts the three tracts dissected in two participants.

##### Hardi

**Figure 4:**
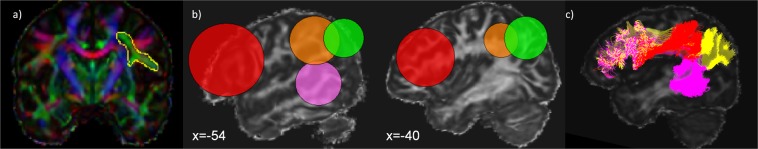
(**a**) Delineation of the fronto-parietal white matter stem at the level of the central sulcus used as ﻿“Any part” inclusion ROI for the three tracts. (**b**) Inclusion ROIs: Frontal sphere (red) for all three tracts, Temporal sphere (fuchsia) for the AF, SMG sphere (orange) for SLF III, ANG sphere (green) for SLF II. (**c**) Final dissections of the three tracts in the left hemisphere for one participant using HARDI.

Because of the much higher number of streamlines and false positives, as well as looping fibers generated by the probabilistic HARDI approach, the protocol was adapted from the method described above. In order to include only the fibers connecting the pars opercularis (area 44) and pars triangularis (area 45) of the IFG and the adjacent ventral premotor cortex (ventral area 6) and the region above the IFG where areas 8A and 9/46 lie an “Either end” volume (sphere, 30 mm radius centered at MNI coordinates [x = −53, y = 27, z = 20] for the left hemisphere and at [x = 49, y = 27, z = 20] for the right hemisphere) was used (see Fig. 4b). The “any part” yellow ROI was still used to exclude streamlines passing outside the green fronto-parietal white matter stem (Fig. 4a) and was hand drawn to ensure that all parts of the green region were included. Occasionally, extra exclusion ROIs had to be used on a case by case basis.

#### Resting state fMRI

In order to examine consistency of resting state data in the human brain with findings from the monkey invasive anatomical tracing studies that made a clear distinction between the SLF III branch originating from the anterior inferior parietal lobule (i.e. the homologue of the supramarginal gyrus) and the SLF II branch originating from the posterior inferior parietal lobule (i.e. the homologue of the angular gyrus), resting state connectivity analyses in the same group of 50 participants were performed.

SPM8 (Wellcome Department of Imaging Neuroscience, London, UK) was used to preprocess the resting-state fMRI data. Standard spatial preprocessing steps were used, including slice-time correction that was applied to the images which were also realigned and resliced, normalized to MNI space, and smoothed with a 6 mm kernel. We performed seed-driven functional connectivity analysis with the CONN software^37,38^. Seed–to-voxel correlations were performed by estimating temporal correlations between the blood oxygen level-dependent (BOLD) signal from our ROIs (seeds) and BOLD signal at every brain voxel.

#### Seeds for ROI-to-ROI and ROI-to-voxel analyses

ROI-to-ROI analysis: We used seeds in the Pars Triangularis (area 45), Pars Opercularis (area 44), Supramarginal Gyrus (SMG, area 40, also known as area PF/PFG), and Angular Gyrus (ANG, area 39, also known as area PG/Opt). The ROIs were defined as in the AAL anatomical atlas^39^ and were created using the WFU pickatlas.

ROI-to-Voxel analysis: We used 5 mm spheres at the following Montreal Neurological Institute (MNI) coordinates (x, y, z): SMG [−60 –41 35], ANG [−41 −71 43], Temporal [−60 −37 2]. The results displayed in Fig. 8 are significant areas of correlated activity for the group using a threshold of uncorrected *p* = 0.001 with a FDR corrected cluster-threshold of *p* = 0.05.

## Results

**Figure 5:**
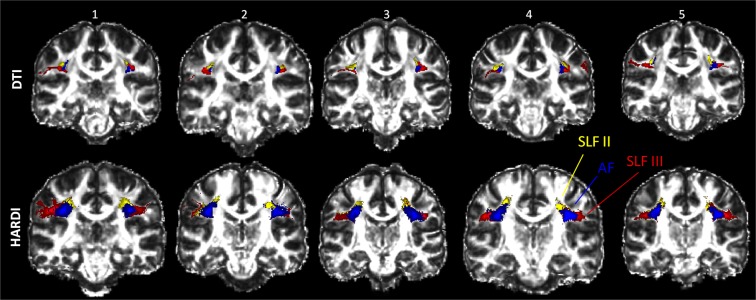
Examples of five different individual brains all showing the same relative positions of the dissected tracts in the coronal view (y = −26). The top row depicts the tracts dissected using the DTI approach and the bottom row the tracts dissected with the HARDI method. SLF III is in red, SLF II in yellow and AF in blue.

The present dissections of the separate tracts provide clear evidence of their relationship to one another in the white matter (see Fig. 5). The position of SLF III (red), which courses lateral to SLF II (yellow) and AF (blue) was observed when using both tractography methods and is also in agreement with the relative position of these tracts in the macaque monkey where they were first identified and separated using gold standard invasive methodology^11,18^. The position of the AF and SLF II in relation to one another is also consistent with the one shown in previous studies^10,13,40^.

**Figure 6:**
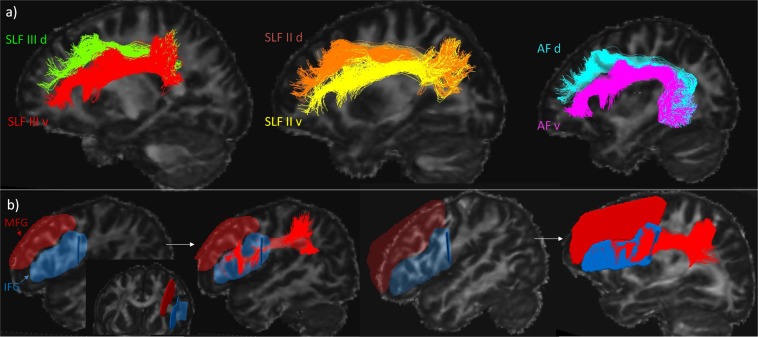
(**a**) Example for one participant of the separation of each tract into dorsal (d; connecting with the MFG; green, orange and cyan for the SLF III, SLF II and AF, respectively) and ventral (v; connecting with the IFG; red, yellow and fuchsia for the SLF III, SLF II and AF, respectively) branches. (**b**) Specifically drawn IFG (blue) and MFG (red) inclusion ROIs for two participants and the example of the ventral SLF III branch linked to the IFG ROI.

With the HARDI method, as shown in Fig. 6a, the dorsal part of each tract that terminates into the middle frontal gyrus (MFG) could be distinguished from the ventral part that is connecting with the inferior frontal gyrus (IFG) using hand-drawn inclusion ROIs around the IFG and the MFG of each participant (Fig. 6b). Separating the dorsal and ventral branches of the reconstructed tracts could be useful in future research to test specific hypotheses about the respective functional roles of the dorsal and ventral connections.

### Success rate by tract and method

The SLF III in the left and right hemispheres were successfully dissected in 100% of participants with both methods. Using the DTI approach, no streamlines were found for the left SLF II in 29% of participants and for the AF, in the right hemisphere, there were no streamlines for 15% of participants. The HARDI method yielded streamlines for the SLF II and AF in both hemispheres for 100% of participants (Table 1).

**Table 1:** Percent of successful dissection of the three different tracts in the participants using either the DTI or the HARDI method.

	Arcuate		SLF II		SLF III	
	Left	Right	Left	Right	Left	Right
DTI	100%	85%	71%	100%	100%	100%
HARDI	100%	100%	100%	100%	100%	100%

### Resting state connectivity

#### ROI-to-ROI connectivity statistics

**Figure 7:**
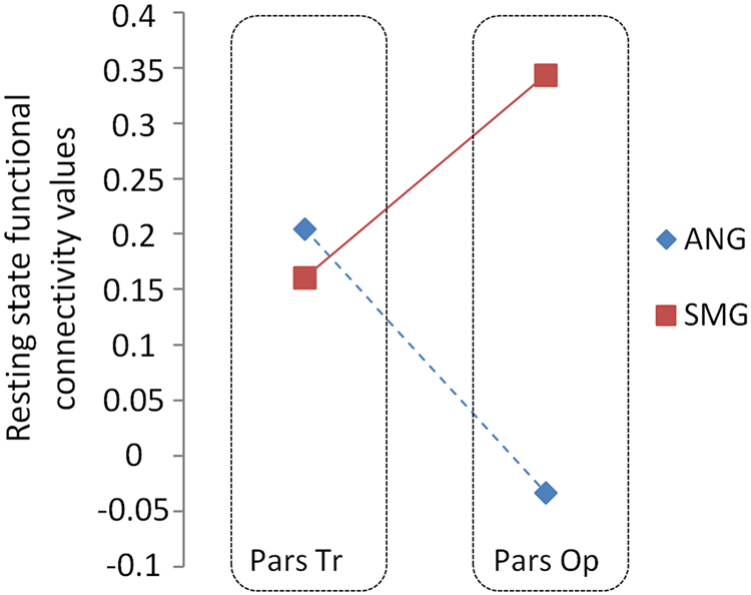
Resting state functional connectivity values from an ROI-to-ROI analysis using two inferior parietal ROIs corresponding to the supramarginal gyrus (SMG; full red line) and the angular gyrus (ANG; dotted blue line) and two inferior frontal ROIs corresponding to pars triangularis (Tr) and pars opercularis (Op). The connectivity values for the four possible fronto-parietal pairs are displayed.

Resting state fMRI connectivity values between the frontal and parietal ROIs were extracted for the group of 50 participants. As shown in Fig. 7, the pars opercularis of the inferior frontal gyrus (where area 44 lies) exhibits significantly greater functional connectivity with the supramarginal than with the angular gyrus (*p* < 0.001). Furthermore, there was significantly greater functional connectivity between the angular gyrus of the inferior parietal cortex and the pars triangularis of the inferior frontal gyrus (where area 45 lies) than the angular gyrus and the pars opercularis (*p* < 0.001, blue dotted line). By contrast, there was significantly greater connectivity between the supramarginal gyrus and the pars opercularis (area 44) than the supramaginal gyrus and the pars triangularis (area 45) (*p* = 0.001, red line). These patterns of resting state connectivity are consistent with predictions from the gold standard anatomical invasive studies in the macaque monkey that demonstrated a distinct pathway (SLF III) from the anterior inferior parietal lobule (i.e. the homologue of the SMG) to area 44 and another distinct pathway (SLF II) from the posterior part of the inferior parietal lobule (i.e. the homologue of the angular region) to area 45^11,35^, and are also consistent with previous resting state functional connectivity data^41,42^.

#### Seed-to-voxel

**Figure 8:**
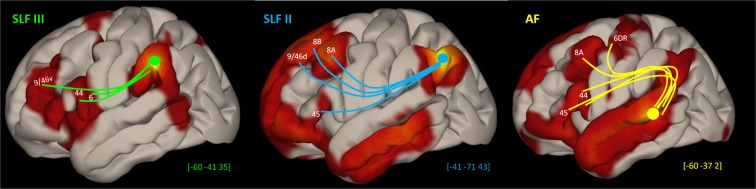
Seed-to-voxel resting state connectivity group results using 5 mm spheres as seeds. The supramarginal seed (green) was placed at MNI coordinates [x = −60, y = −41, z = 35], the angular seed (blue) at [x = −41, y = −71, z = 43], and the temporal seed (yellow) at [x = −60, y = −37, z = 2]. Significance threshold of *p*-uncorrected height of 0.001 and FDR corrected cluster-threshold of *p* = 0.05. Schematic representation of each tract and their frontal targets are displayed.

To investigate and visualise the distinct frontal areas where the resting-state activations correlated with the supramaginal (SMG) and angular (ANG) regions of the inferior parietal lobule, we placed 5 mm spheres in the posterior temporal, SMG and ANG regions. The results displayed in Fig. 8 are significant areas of functionally correlated activity for the group. The SMG seed shows significant functional connectivity with area 44 on the pars opercularis and also cortex just above the inferior frontal sulcus where area 9/46v lies at the junction with area 45^43^. By contrast, the ANG seed shows connectivity with the pars triangularis where area 45 lies^44^. Of note, the SMG connectivity in the frontal lobe outside Broca’s region, i.e. with area 9/46v on the lower part of the middle frontal gyrus just above the inferior frontal sulcus and the anterior ventral premotor region (area 6VR), is consistent with the invasive tract tracing investigation in the monkey that first discovered these connections and named them as SLF III^11^ and also confirms earlier resting state fMRI data^41,42^ of a functional correlation between the SMG and cortex on the most ventral part of the middle frontal gyrus. Furthermore, there is an interesting detail first detected in the macaque monkey invasive anatomical studies regarding the connectivity of the anterior inferior parietal lobule (homologue of SMG) with the frontal cortex via SLF III. The SMG can be divided into anterior (area PF) and posterior (area PFG) parts (see^36^, pp. 124–130 for a description of the differences in cytoarchitecture). Although both parts are connected with the frontal cortex via the SLF III, precise macaque monkey invasive anatomical studies have shown that the stronger connection to area 44 originates from area PFG (i.e. the homologue of the posterior SMG)^35,45^. This difference has also been observed in resting-state connectivity in the human brain. When the seed was placed in the anterior SMG, the connectivity was primarily with ventral area 6 and area 9/46v, but when the seed was placed in the posterior SMG, the connectivity was also strong with area 44 on the pars opercularis (see Supplementary Fig. S1). Similarly, the strongest connectivity with area 45 originates from area PG in the anterior angular gyrus, again consistent with macaque monkey studies (see^45^).

Overall, as can clearly be seen in Fig. 8, the SMG and ANG seeds show opposite resting state connectivity patterns within the frontal lobe, consistent with gold standard anatomical knowledge obtained in invasive macaque monkey experiments. Moreover, a seed placed at the junction of the posterior parts of the superior and middle temporal gyri from where the AF originates, clearly shows functional connectivity with areas 44 and 45 (ventral branch of AF) as well as area 8A and the adjacent rostral part of dorsal area 6 (6DR).

## Discussion

Using critical information from macaque gold standard invasive anatomical tract tracing, as well as earlier *in vivo* diffusion MRI findings and resting state connectivity data in the human brain, we developed refined protocols to dissect the classical arcuate fasciculus (AF), namely the fiber tract *arching around the end of the Sylvian fissure* to connect the posterior temporal region with specific areas of the frontal cortex. In addition, we were able to distinguish the AF from the adjacent superior longitudinal fasciculus (SLF) branches II and III, which course parallel to the AF in the parieto-frontal white matter (e.g., under the central sulcus) and connect the angular gyrus in the posterior IPL (SLF II) and the supramarginal gyrus in the anterior IPL (SLF III) with distinct parts of the lateral frontal cortex. This information is critical for future studies that may wish to correlate behavioral performance and other functional indices with anatomical properties of these distinct pathways. Another major contribution of the present study is the demonstration, for the first time, of a dorsal branch of the AF that terminates in the *posterior dorsolateral* frontal region in dorsal area 8A and the adjacent rostral part of dorsal area 6, clearly distinct from the traditionally described and reconstructed fibers of the AF to the IFG that is here referred to as the ventral branch. The dorsal branch of the AF was first demonstrated in invasive tract tracing studies in the monkey^18^ but had not previously been reported in the human brain. The macaque monkey studies had clearly shown that, as expected, the arching fibers of the AF link the posterior temporal region with the inferior frontal region, but that, in addition, there was a distinct dorsal branch of these fibers that links the *posterior dorsolateral* frontal region (i.e. area 8A and the adjacent rostral premotor cortex) with the posterior temporal region. The dorsal branch of the AF would thus permit interaction, in the human brain, between the high level attentional control provided by area 8A with posterior temporal auditory processing, such as musical and other nonverbal processing in the right non-dominant hemisphere, and attentional interaction with Wernicke’s area in the language dominant hemisphere which is often disrupted after AF lesions [see^4^]. The study of this dorsal frontal branch of the AF will also permit examination of functional interaction between the posterior temporal lobe and the dorsal premotor zone that controls the hand/arm musculature necessary, perhaps, for gestural communication in the language dominant hemisphere.

Although these three distinct tracts, i.e. the AF, SLF II, and SLF III, have traditionally been considered in relation to various aspects of language processing in the language dominant hemisphere of the human brain, they are *not* just language processing tracts, especially in the non-dominant right hemisphere of the human brain. For example, the posterior inferior parietal region in the right non-dominant hemisphere is known to play a major role in spatial processing. In an investigation of the anatomical correlates of chronic unilateral neglect (without hemianopia) in patients with lesions in the right hemisphere, the maximal site of lesion overlap was observed in the superior longitudinal fasciculus in the white matter of the anterior inferior parietal lobule^46^. Thus, a parieto-frontal disconnection resulting from damage to the superior longitudinal fasciculus may result in a failure to attend to spatial information in the contralateral visual field yielding unilateral neglect.

The relative roles of SLF II vs SLF III and AF can only be explored if these pathways can be dissected separately, which was one of the major achievements of the present study. Accurate dissection and clear separation of these distinct tracts is essential for understanding their specific functional contributions and important for future research aimed at investigating pathological conditions that affect these pathways and documenting their specific effects on neuropsychological performance. The distinct reconstruction of these pathways is also critical for future neuroimaging studies attempting to correlate properties of these tracts with various aspects of cognitive and motor performance. For example, although conduction aphasia is often referred to as a syndrome following AF lesions in the language dominant hemisphere [e.g.,^3^], the extent to which the deficit is associated with AF lesions as opposed to SLF II and/or SLF III lesions or a combination of damage to these three distinct pathways remains to be established. Similarly, further research in the human brain is necessary to refine our knowledge of the functional roles of the SLF. Separating the SLF II and III into dorsal (connections above the IFG) and ventral (connections to the IFG) branches is also relevant. For example, in the language dominant left hemisphere, the dorsal branch of SLF III connecting mostly with area 9/46v (which is part of a functional monitoring system in working memory^47^) is likely to be involved in the monitoring of phonological and speech output information, while its ventral branch that connects with ventral area 6 and area 44 may play a role more specifically related to phonological processing during speech production^36,48^. The SLF II is linking the posterior part of the inferior parietal lobule (angular gyrus) that is critically involved in the processing of visual and multisensory spatial information with area 8A, namely the dorsolateral prefrontal hub for top-down attentional control and is thought to be a special pathway for attentional allocation and, in the language dominant hemisphere, is known to play important roles in reading^49^. By contrast, the SLF III is hypothesised to be involved in articulation and phonological processing [see ^48^]. Indeed, there is already functional neuroimaging data that area 44, which is connected via the SLF III with the supramarginal gyrus, shows increased activity in relation to phonological processing^50^.

In line with monkey investigations using precise invasive anatomical tracers, previous resting state connectivity studies reported stronger connectivity of the angular gyrus with the pars triangularis of the IFG where area 45 lies, and stronger functional connectivity of the supramarginal gyrus with ventral area 6 on the precentral gyrus and area 44 on the pars opercularis^41,42,51^ and the present resting state connectivity analysis using inferior parietal seeds agrees with this pattern. Note, however, that the resting state “connectivity” is fundamentally correlations of activation states and anatomical studies, such as the present one, are necessary to dissect the white matter fasciculi that permit these interactions.

Specific anatomical landmarks, such as sulci and gyri, were used to place inclusion and exclusion regions of interest (ROIs) based on the best available evidence regarding the cortical areas that are connected by each tract. For example, previous protocols designed to dissect the AF acknowledged that the fibers dissected extended outside of the classical posterior temporal region^52^. In the present study, we focussed on dissecting the arching fibers that originate from the posterior temporal region but not restricting the origin of the AF only to the superior temporal gyrus, as we also included the cortex within the adjacent superior temporal sulcus and the posterior part of the middle temporal gyrus that are known to contribute fibers. We also designed our protocol to separate the fibers at the ambiguous temporo-parietal junction in a consistent and reproducible manner in order to distinguish the AF from the SLF II and III which in earlier protocols were not treated as distinct pathways separate from the AF^6,7,9^.

In explaining their ROI placement, Wakana *et al*.^8^ instruct that the temporal ROI be placed at the level of the anterior commissure, which, depending on the angle of acquisition of the images in the scanner, can be too low and not include all fibers in the posterior superior temporal gyrus, near the parietal junction. The same could occur with other protocols that place the temporal ROI with no specification in terms of axial slice^9^. In reconstructing the AF, we suggest the use of specific sulci and gyri as landmarks for ROI placement to ensure that only the fibers originating below the “dplf” (see Figs. 1–3) are included, i.e. only the posterior temporal *arching* fibers. Moreover, in some previous tractography protocols, when dissecting the parieto-frontal fibers, no distinction was made between fibers from different parts of the inferior parietal lobule and, therefore, the SLF II and SLF III pathways were inevitably mixed together, although both precise anatomical tract tracing in the macaque monkey brain and functional resting state connectivity in the human brain clearly indicate that these are two distinct parallel pathways connecting distinct areas of the frontal cortex with the supramarginal (SLF III) and the angular (SLF II) regions of the inferior parietal lobe. More recent studies^12,13^ did separate the SLF into its branches from the parietal lobe but restricted the connections from the angular gyrus (SLF II) to the middle frontal gyrus, excluding its connection to area 45 and restricted the connections from the supramarginal gyrus (SLF III) to the inferior frontal gyrus excluding the fibers terminating in the posterior part of ventral middle frontal gyrus where area 9/46v lies. The present study succeeds in separating these two distinct branches of the superior longitudinal fasciculus, i.e. the SLF II from the angular gyrus and the SLF III from the supramarginal gyrus, in agreement with the precise monkey tract tracing and earlier research in the human brain (see above) and also clearly separates these parieto-frontal tracts from the temporo-frontal tract *arching around the end of the Sylvian fissure*, namely the arcuate fasciculus (AF) by the use of precise ROIs based on monkey tracing and human resting state connectivity data.

The present investigation also demonstrates that the tractography algorithm used can affect the outcome in terms of success in dissecting the tracts, especially the SLF II in the left hemisphere. In the latter case, the HARDI approach proved to be significantly more successful than DTI in which more than one third of the participants had no streamlines connecting the angular gyrus to the frontal lobe. The dissection of the AF in the right hemisphere was also problematic using the tensor model and was “absent” in some participants. This observation is in line with Bain *et al*.^33^ who showed that the laterality indices of the AF are affected by the protocol used and, therefore, drawing conclusions about the right AF in comparison to the left AF requires caution: the method used to reconstruct the AF must be considered in laterality studies.

## Data availability

The data that support the findings of this study are available from the corresponding author upon request.

## Supplementary information

Supplementary Information.
