# Supplementary material for: Dissociating the white matter tracts connecting the temporo-parietal cortical region with frontal cortex using diffusion tractography
Source: Sci Rep. 2020 May 18;10:8186. doi: 10.1038/s41598-020-64124-y (PMC7235086; doi:10.1038/s41598-020-64124-y)
Supplement: Supplementary file 1 — Supplementary Information. [file 41598_2020_64124_MOESM1_ESM.docx]

**Supplementary Information**:

**Dissociating the white matter tracts connecting the temporo-parietal cortical region with frontal cortex using diffusion tractography**

Elise B. Barbeau, Maxime Descoteaux & Michael Petrides


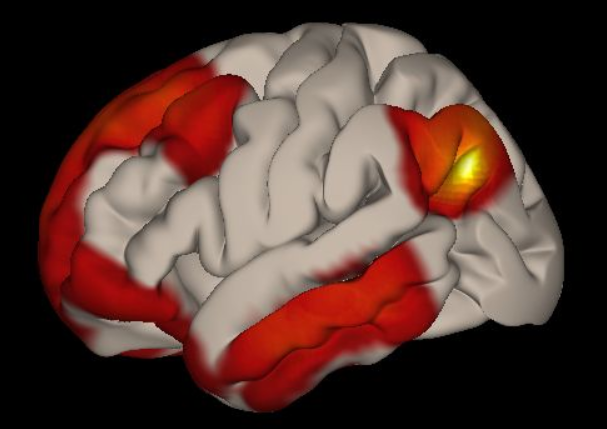


8A

8B

9/46d


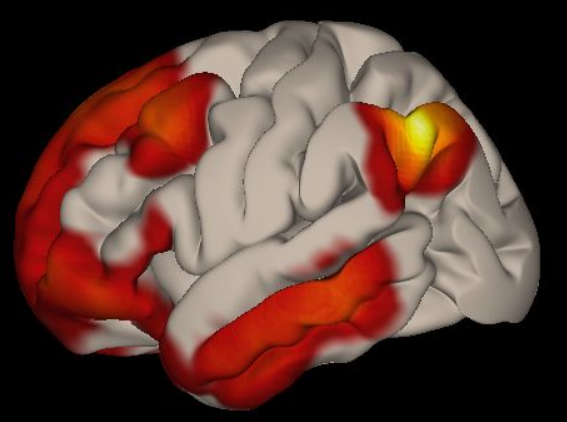


8A

8B

9/46d

45


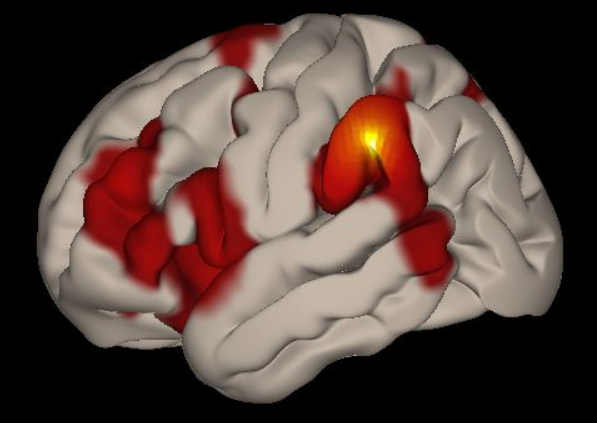


44

9/46v

9/46v

al

Op


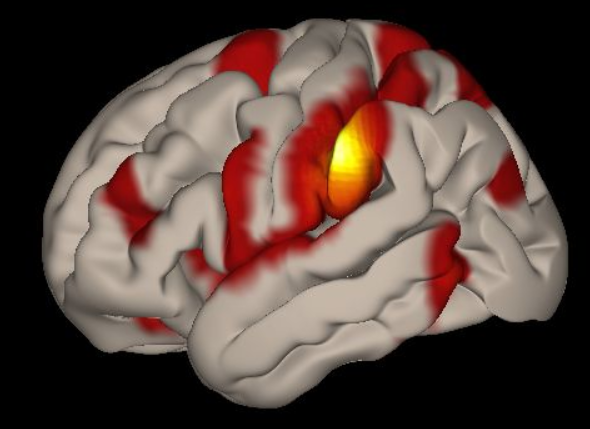


9/46v

6VR

aI

ScOp

aANG

pANG

pSMG

aSMG

6VR

aI

ScOp

**Supplementary Figure S1.** Different seed-to-voxel resting state connectivity patterns from anterior (a) and posterior (p) areas of the supramarginal gyrus (SMG) and angular gyrus (ANG). The upper panel shows that both aSMG (area PF) and pSMG (area PFG) show correlated activations with area 9/46v, anterior Insula (aI), rostral area 6 ventral (6VR) and the subcentral opercular zone (ScOp), but the correlation with area 44 is mostly from the pSMG seeds (area PFG). The lower panel shows that both aANG and pANG show correlated activations with area 9/46d and areas 8A and 8B in the dorsolateral frontal cortex but that the connection with area 45 on the inferior frontal gyrus is mostly with the aANG (area PG), rather than the pANG (area Opt). These findings are in agreement with gold standard macaque monkey anatomical connectivity data. Note that the labeled areas in the lateral frontal cortex are known to be directly connected via SLF III with SMG and via SLF II with ANG. But note also that resting-state connectivity also shows additional frontal areas (not labeled) that are correlated with temporal cortex (via the temporo-frontal extreme capsule fasciculus), as well as via local frontal interconnectivity. Resting-state connectivity simply shows correlated activity between areas and does not imply direct monosynaptic anatomical connections via a specific fasciculus.

.
